# Emotional visual mismatch negativity: a joint investigation of social and non-social dimensions in adults with autism

**DOI:** 10.1038/s41398-020-01133-5

**Published:** 2021-01-05

**Authors:** Klara Kovarski, Judith Charpentier, Sylvie Roux, Magali Batty, Emmanuelle Houy-Durand, Marie Gomot

**Affiliations:** 1 UMR 1253 iBrain, Université de Tours, Inserm, Tours, France; 2 Hôpital Fondation Adolphe de Rothschild, Paris, France; 3 Université de Paris, CNRS, Integrative Neuroscience and Cognition Center, 75006 Paris, France; 4 Université de Toulouse, CERPPS, Toulouse, France; 5 CHRU de Tours, Centre Universitaire de Pédopsychiatrie, Tours, France

**Keywords:** Neuroscience, Autism spectrum disorders

## Abstract

Unusual behaviors and brain activity to socio-emotional stimuli have been reported in Autism Spectrum Disorder (ASD). Atypical reactivity to change and intolerance of uncertainty are also present, but little is known on their possible impact on facial expression processing in autism. The visual mismatch negativity (vMMN) is an electrophysiological response automatically elicited by changing events such as deviant emotional faces presented among regular neutral faces. While vMMN has been found altered in ASD in response to low-level changes in simple stimuli, no study has investigated this response to visual social stimuli. Here two deviant expressions were presented, neutral and angry, embedded in a sequence of repetitive neutral stimuli. vMMN peak analyses were performed for latency and amplitude in early and late time windows. The ASD group presented smaller amplitude of the late vMMN to both neutral and emotional deviants compared to the typically developed adults (TD) group, and only the TD group presented a sustained activity related to emotional change (i.e., angry deviant). Source reconstruction of the vMMNs further revealed that any change processing elicited a reduced activity in ASD group compared to TD in the saliency network, while the specific processing emotional change elicited activity in the temporal region and in the insula. This study confirms atypical change processing in ASD and points to a specific difficulty in the processing of emotional changes, potentially playing a crucial role in social interaction deficits. Nevertheless, these results require to be further replicated with a greater sample size and generalized to other emotional expressions.

## Introduction

Autism Spectrum Disorder (ASD) is a neurodevelopmental disorder characterized by two main categories of symptoms, the first including disturbances in the socio-emotional domain and the second including restricted interests, stereotyped behaviors, and atypical sensory responsiveness^1^. Difficulties in social interactions have been associated with atypical social cognition and emotional processing, while signs observed in the second clinical dimension have often been related to intolerance of uncertainty, a dispositional trait implying a reduced adaptation under unpredictable conditions^2^. Though the combination of both social difficulties and repetitive behaviors is required for ASD diagnosis, the interplay between these two clinical dimensions remains barely studied.

In line with difficulties for ASD in the social domain, faces represent particularly challenging stimuli to apprehend. Faces are socially relevant and convey rapid information about others’ intentions. Facial expressions provide additional important cues triggering emotion-related attentional mechanisms. Because of its social value, detecting changes in facial expressions is a fundamental requisite to avoid danger and allows adaptation to others’ reactions. In autism, impaired emotional face recognition has repeatedly been reported^3,4^, in line with atypical amplitude and/or latency of event-related potentials (ERPs) or patterns of brain activity in response to both static and dynamic emotional faces^3,5–7^. Symmetrically, sensory particularities and intolerance of uncertainty in ASD might relate to atypical processing of changes in sensory information^8–12^. In the visual domain, automatic change detection responses can be investigated via oddball paradigms allowing to record the visual mismatch negativity (vMMN)^13^. The vMMN is a negative component representing the automatic neural mechanism involved in the processing of unexpected information. vMMN results from the subtraction of a standard stimulation from a deviant stimulation and it is usually observed in early latency range peaking around 100–250 ms post stimulus and has been observed in response to different deviancies such as color, shape, or motion^14^. vMMN has also been identified in response to complex stimuli such as facial expressions peaking within a large time window including one or two deflections depending on the study (100–500 ms, refs. ^15–20^). In a previous study on typically developed adults (TD) using a tightly controlled paradigm, we also showed that deviant expressions elicited an early and a late vMMN^18^. Interestingly, while both neutral and emotional deviants elicited vMMN peaks, only the emotional deviancy evoked a late sustained specific response following the later peak.

Although several studies have investigated vMMN in response to simple stimuli in individuals with psychiatric conditions such as schizophrenia (see for a review, ref. ^21^), little research has been conducted on autism^9,22–26^. Using deviant and novel geometric stimuli during an oddball sequence, it has been shown that children and adults with autism displayed earlier and smaller vMMN compared to controls^9,23^, suggesting unusual brain reactivity to visual changes. Few studies have specifically investigated facial expression vMMN in psychiatric conditions^27–29^. One study on major depression disorder showed that early vMMN was reduced and that the later activity was absent in the clinical group^27^. Similar results were also observed in schizophrenic patients who presented smaller amplitude to fearful and happy deviant faces^28^. While no studies have investigated vMMN to facial expressions in autistic individuals, one previous work has measured autistic personality traits assessed via the Autism-Spectrum Quotient (AQ) in TD adults, revealing a positive correlation between the AQ scores and the emotional vMMN amplitude. This suggests that vMMN component is sensitive to autistic traits and that greater autistic traits are associated with smaller sustained vMMN mean amplitude (i.e., more positive, ref. ^30^). Despite growing evidence showing that deficits are not restricted to the social domain, few studies have jointly investigated socio-emotional difficulties with intolerance to change in ASD, by combining both diagnostic dimensions^31^. Accordingly, emotional vMMN is a particularly suitable tool to explore the brain processes at the interplay between the two diagnostic criteria in autism. By using a highly controlled paradigm, the present study aimed at investigating automatic change detection to emotional faces in adults with ASD. To this end, both oddball and equiprobable sequences have been presented to participants allowing to control for neural habituation and to low-level features^32,33^. Both an emotional (i.e., angry face) and a neutral deviant were presented to investigate the automatic emotional change response^18^. In accordance with the literature and clinical observations in individuals with ASD, we expected (i) a disruption of the automatic change detection and (ii) a lack of modulation of the change detection process according to the emotional value/nature of the deviant stimulus. Conversely, in the TD group an emotion-specific response to angry deviants should be recorded, as previously shown^18^.

## Material and methods

### Participants

Thirty-seven participants completed the electroencephalographic (EEG) task. An original sample of 20 adults with ASD completed the task; however, 3 participants were excluded from the group analyses because of poor EEG recording quality (e.g., noisy signal, few averaged trials). Seventeen adults with ASD (mean age ± standard deviation: 25.7 ± 6.4; 3 females) were matched by chronological age to 17 TD adults (mean age: 26.3 ± 6.8; 3 females, age group comparison: *t*(32) = 0.26, *P* = 0.80). ASD participants were evaluated by an experienced clinical team at the Autism Resource Centre of Tours and were diagnosed according to the Diagnostic and Statistical Manual of Mental Disorders criteria and by using the Autism Diagnostic Observation Schedule, Second Edition and/or the Autism Diagnostic Interview-Revised scales^1,34,35^. Only one participant was medicated with neuroleptic treatments, which did not influence group data. None of the TD participants reported any developmental difficulties in language or sensorimotor acquisition. For all participants, no disease of the central nervous system, infectious/metabolic disease, epilepsy, or auditory/visual deficit was reported.

Intellectual Quotients (verbal and performance IQs) were obtained using the Wechsler Intelligence Scale for Children Fourth Edition^36^. An estimation of verbal and performance IQs was performed in the TD group using four selected subtests (Vocabulary, Similarities, Block Design, and Matrix). Two-tailed *t* tests revealed that groups differed on both non-verbal (ASD: 97.1 ± 23; TD: 112.1 ± 11.4; *t*(32) = −2.42, *P* = 0.02) and verbal IQ (ASD: 101.2 ± 23.6; TD: 117.9 ± 14; *t*(32) = −2.51, *P* = 0.04); however, none of the participants presented a developmental delay (IQ < 70).

Written informed consent for the experiment was collected from all participants. The protocol was approved by the local Ethics Committee according to the ethical principles of the Declaration of Helsinki (Clinical trial: NCT02160119).

### Stimuli and procedures

**Fig. 1:**
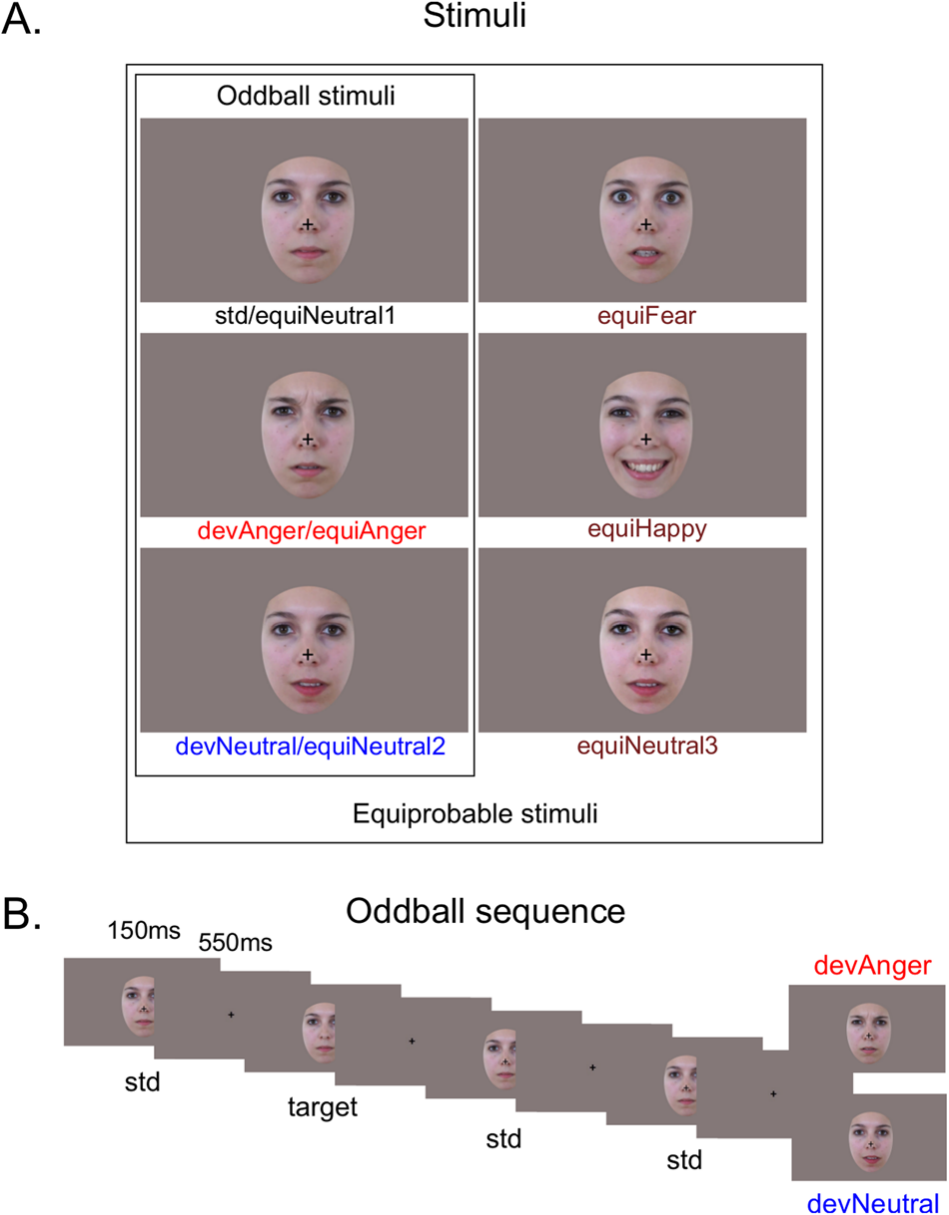
Stimuli and example of oddbal sequence. **A** Illustration of the stimuli used in the oddball sequence (*N* = 3) and in the equiprobable sequence (*N* = 6). **B** Example of the oddball sequence showing the time course of the task. Stimuli were presented for 150 ms, followed by the central cross presented for 550 ms (SOA = 700 ms). Target consisted of a standard face stimulus with no cross (participants had to detect the disappearance of the central cross).

The procedure and stimuli used in the present study were the same as previously reported^18^. Six photographs of the same actress (Fig. 1A) were presented in two sequences: an oddball and an equiprobable sequence (Fig. 1A). In the latter, stimuli had equal probability of occurrence. This sequence was presented to control for neural habituation due to stimulus repetition in oddball sequences (standard stimulus)^32,33,37^. Accordingly, controlled vMMN was performed by subtracting the evoked response of the deviant stimulus (in the oddball sequence) from the same stimulus presented in the equiprobable sequence. This allows reducing low-level feature effects and revealing genuine automatic change detection responses^38^.

In the oddball sequence, a neutral expression was presented as the standard stimulus (std; probability of occurrence *P* = 0.80). Photographs of the same actress expressing anger or with a different neutral expression were presented as the emotional deviant (devAnger, *P* = 0.10) and the neutral deviant (devNeutral, *P* = 0.10) respectively. In the equiprobable sequence, six stimuli were presented: the three stimuli of the oddball sequence and three other facial expressions. Altogether the equiprobable sequence included angry (the same stimulus as the angry deviant: equiAnger), fearful and happy faces (equiFear and equiHappy, respectively), and three neutral faces (equiNeutral1: same as the standard stimulus, equiNeutral2: same as the neutral deviant, and equiNeutral3) presented pseudo-randomly (*P* = 0.16 each), avoiding immediate repetition. Responses to the equiFear, equiHappy, and equiNeutral3 stimuli were not further analyzed as these stimuli were added to respect the design of the equiprobable sequence. Stimuli were previously behaviorally validated for their emotional significance and arousal on an independent group of participants; for details, see ref. ^18^. Deviants and the standard stimuli were normalized for luminance and contrast.

Participants sat comfortably in an armchair 120 cm from the screen. By using the Presentation® software, stimuli were presented in the central visual field (visual angle: width = 5.7°, height = 8.1°) for 150 ms with a 550- ms inter-stimulus interval (Fig. 1B). The short presentation of the stimuli allowed preventing from saccadic movements toward the eyes or mouth region. The oddball sequence comprised 1575 stimuli and the equiprobable sequence 924 stimuli. Total recording time lasted 30 min. Subjects were asked to focus on a concurrent visual task: target stimuli consisted of a face stimulus in which a black fixation cross on the nose, otherwise present, disappeared. Participants were instructed to look at the fixation cross and to press a button as quickly as possible when the cross disappeared. Targets occurred on neutral standards in the oddball sequence and on any stimulus in the equiprobable sequence (*P* = 0.05). All subjects were monitored with a camera during the recording session to ensure compliancy to the task.

### Behavioral analysis

Accuracy and false alarms (FAs) during the target detection task were analyzed and the sensitivity index, *d*’ = *z*-score (% correct responses) − *z*-score (FAs) was measured to evaluate the degree of attention of the participants. A *t* test was performed to compare groups.

### EEG recording

EEG data were recorded using a 64-channel ActiveTwo system (BioSemi^®^, The Netherlands). Two electrodes were applied on the left and right outer canthi of the eyes and one below the left eye to record the electrooculographic activity. An additional electrode was placed on the tip of the nose for offline referencing. During recording, impedances were kept <10 kΩ. EEG signal was recorded with a sampling rate of 512 Hz and filtered at 0–104 Hz.

### Pre-processing

A 0.3-Hz digital high-pass filter was applied to the EEG signal. Ocular artifacts were removed by applying Independent Component Analysis (ICA) as implemented in EEGLab. Blink artifacts were captured into components that were selectively removed via inverse ICA transformation. Thirty-two components were examined for artifacts and one or two components were removed in each subject. Muscular and other recording artifacts were discarded manually. EEG data were recorded continuously and time locked to each trial onset. Trials were extracted over a 700-ms analysis period, from 100 ms pre-stimulus to 600 ms post-stimulus. ERPs were baseline corrected and digitally filtered with a low-pass frequency cut-off of 30 Hz.

**Fig. 2:**
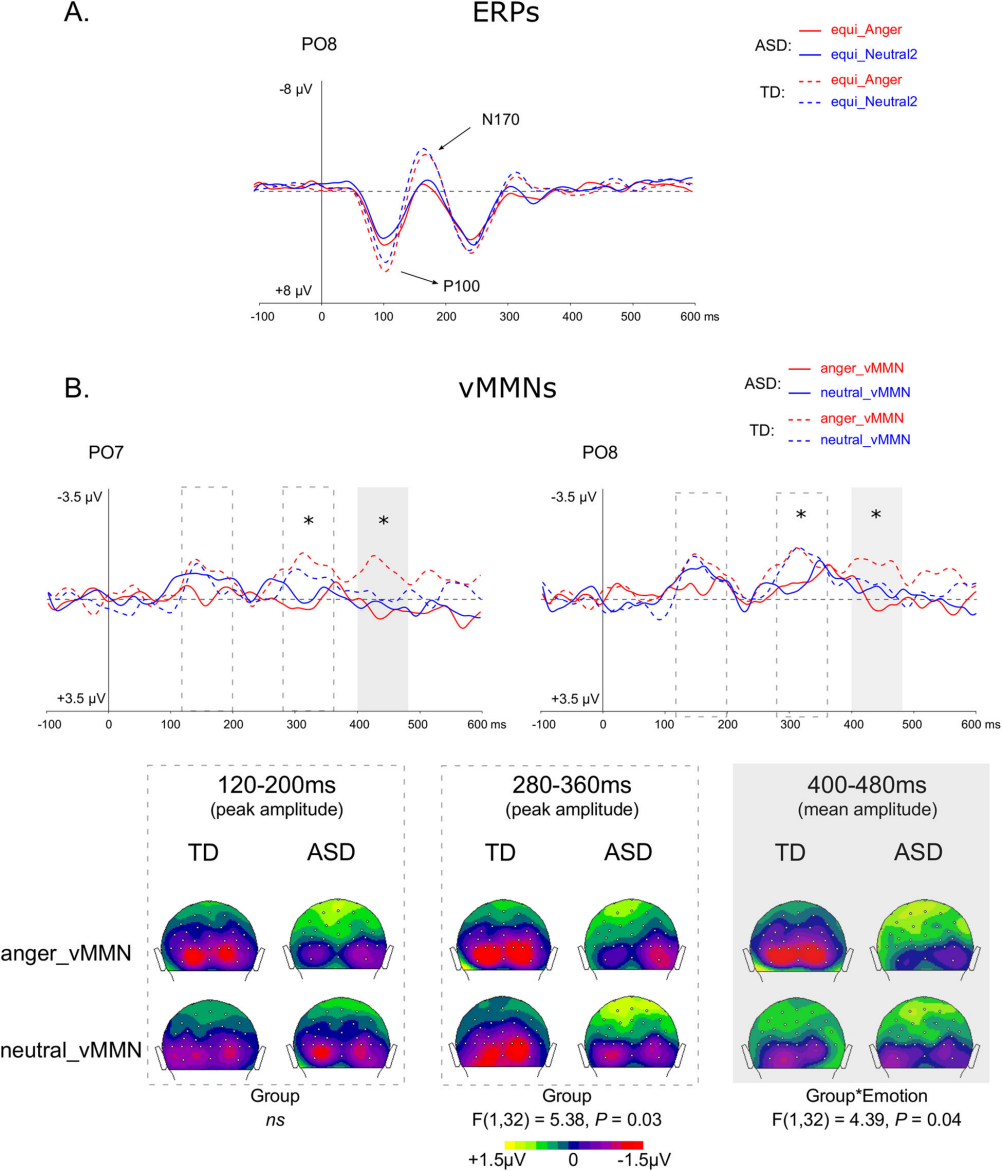
Sensory ERPs and vMMNs for each condition and group over parieto-occipital electrodes. **A** Grand-average ERPs at PO8 elicited by emotional (i.e., anger, red lines) and neutral (blue lines) stimuli presented in the equiprobable sequence for the ASD (plain lines) and TD (dotted lines) groups. P1 and N170 components are indicated by arrows. **B** On the top, grand-average vMMN at PO7 and PO8 elicited by emotional (angry, red lines) and neutral (blue lines) deviants for the ASD (plain lines) and for TD (dotted lines) groups. Gray rectangles represent the time windows in which analyses were performed (peak analyses for 120–200 ms and 280–360 ms and 400–480 ms for mean amplitude). On the bottom, 2D scalp topographies (back view) showing mean activity in selected time windows for analyses.

The first three trials of a sequence, as well as trials occurring after deviant or target stimuli were not included in averaging. Each ERP was computed by averaging all trials of each stimulus type (see Fig. 2A) from the oddball sequence (std, devAnger, devNeutral) and from the equiprobable sequence (equiAnger, equiNeutral2). For each stimulus of interest, the average of artifact-free trials was, for the TD group: 679 ± 113 (std), 128 ± 17 (devAnger), 128 ± 19 (devNeutral), 123 ± 15 (equiAnger), and 121 ± 21 (equiNeutral2); for the ASD group: 691 ± 84 (std), 127 ± (devAnger), 128 ± 17 (devNeutral), 127 ± 15 (equiAnger), and 123 ± 15 (equiNeutral2). Groups did not differ in the number of averaged stimuli regardless of the stimulus category (all *P* > 0.57).

Responses were analyzed and compared with the ELAN software^39^. vMMNs were calculated as ERPs to devAnger and ERPs to devNeutral stimuli minus the responses elicited by the same emotional (equiAnger) and neutral (equiNeutral2) stimuli, respectively, presented in the equiprobable sequence (i.e., anger vMMN = devAnger − equiAnger; neutral vMMN = devNeutral − equiNeutral2); see refs. ^18,33^. Group grand average difference waveforms across participants were examined to establish deflections of interest. Group comparisons were performed on specific brain activities related to emotion deviants and neutral deviants.

### Statistical analyses

#### Event-related potentials

To investigate sensory ERP responses, the P100 and N170 peak latency and amplitude in response to anger and neutral stimuli presented in the equiprobable sequence (namely, equiAnger and equiNeutral2) were analyzed. For the P100, peaks were measured individually in the 80–140 ms time window (see Fig. 2A). A repeated-measure analysis of variance (ANOVA) included Group (TD, ASD) as between-subject factor and Emotion (Anger, Neutral) and Electrode (O1, O2) as within-subject factors. A similar analysis was carried out for the N170 but on PO7 and PO8 electrodes in the 140–200 ms time window.

#### Visual mismatch negativity

For each condition (i.e., anger and neutral vMMN) and each group, vMMN amplitudes were compared to baseline at each electrode and each time point. Then, based on previous emotional face vMMN studies and on visual inspection of averaged vMMN responses in both groups, three time windows were selected to measure early and late peak latency (80–180 ms and 220–400 ms, respectively) and amplitude and mean amplitude (400–480 ms) in all participants. These time windows were similar to responses previously observed in neurotypical individuals and allowed comparing the clinical groups.

##### vMMN peak analyses

Peak latency and amplitude were measured over occipital and parietal sites in two time windows (80–180 ms and 220–400 ms) as the largest negativity within the range for each participant to investigate early and late vMMNs, respectively.

##### Sustained vMMN mean amplitude

Mean amplitude was measured in a selected time window (400–480 ms) in each participant, as it rather reflects sustained responses compared to tighter peak activity (see ref. ^18^).

##### vMMN statistical analyses

Peak (latency, amplitude) and mean amplitude analyses ANOVA were performed on six posterior electrodes: O1, O2, PO7, PO8, P7, and P8 with Group (TD, ASD) as between-subject factor and Emotion (Anger, Neutral), Electrode (Occipital, Parieto-occipital, Parietal), and Hemisphere (Left, Right) as within-subject factors.

For each ANOVA, Bonferroni post hoc comparisons were performed when necessary. For significant results, the effect sizes are shown as *η*^2^_p_. Levene tests were performed to control for group variance if group effects were found.

As group differences were observed on both verbal and performance IQs, to control that IQ did not affect brain responses, analyses of correlation between vMMN and both verbal and performance IQ were performed in each group separately. Correlations are reported whether significant differences were observed.

### Permutation analyses

For each condition, a permutation test in the 50–550 ms time window was performed to compare between groups activity recorded over all the electrodes. Results were corrected for multiple comparisons across time^40^. These analyses provide supplementary information on group differences by confirming peak analyses or proving topographical findings.

### Source analysis

We performed source reconstruction to estimate the anatomical location of electric sources that could explain ERP data. We used the Curry 8 software (Compumedics USA, Inc.) which implemented the methodology proposed by Wagner et al. Distributed source analysis was performed with the sLORETA method (standardized low-resolution brain electromagnetic tomography) for current density reconstruction (CDR) and a realistically shaped three-compartment boundary element method as head model^41^. CDR results for each subject, each emotion (Anger, Neutral), and each condition (Deviant, Equiprobable) were analyzed using the CDR SnPM algorithm (CDR–Statistical non-Parametric Mapping), which is able to extract brain locations through time with significantly different activations between Groups, Emotions, and Conditions, including both main effects and interactions^42^.

For each group, sLORETA images for ERP sources to the emotional vs. neutral stimuli (Emotion effect) in the deviant vs. equiprobable conditions (Condition effect) were compared statistically with a voxel-by-voxel method. Multiple comparisons were corrected by a randomized normalized test based on SnPM. The Group effect (TD vs. ASD) and the Group-by-Emotion interaction of the current sources of emotional and neutral vMMN were performed in the time window between 50 and 500 ms post stimuli onset. The voxels with significant differences (*P* < 0.05) were projected in specific brain regions.

## Results

### Behavioral results

The ASD group presented smaller *d*’ values compared to the TD group (ASD: *d*’ = 3.4 ± 1.5; TD: *d*’ = 4.7 ± 0.75; *t*(32) = 3.23, *P* = 0.003). This was due in particular to four participants in the ASD group presenting a poor task performance (*d*’ < 2.0). The other participants performed the task well (ASD: *d*’ = 4.0 ± 1.0, *N* = 13). The recordings of the four subjects underscoring on the behavioral task were visually inspected to ensure that these participants looked at the screen during the task. Accordingly, both P1 and N170 components were present in these participants suggesting basic face processing and compliance to the task regardless of the poor performance. Similarly to verbal and performance IQ correlations, *d*’ was introduced in correlation analyses together with vMMN parameters, whenever previous electrophysiological statistics revealed significant group differences.

### Event-related potentials

ERPs over PO8 electrode are displayed in Fig. 2A. The results revealed no significant effects on the P100 latency (*P* > 0.21). However, a main effect of Group showed that the ASD group presented a smaller amplitude of the P100 than the TD group (*F*(1,32) = 5.09, *P* = 0.03, *η*^2^_p_ = 0.137). A significant main effect of Emotion (*F*(1,32) = 12.88, *P* = 0.001, *η*^2^_p_ = 0.287) was due to angry faces eliciting a greater P100 compared to neutral face. However, the interaction between Group and Emotion was also significant (*F*(1,32) = 5.78, *P* = 0.02, *η*^2^_p_ = 0.153), explained by the fact that the emotional modulation of P1 amplitude was present in the TD group only (*P* = 0.001) and that groups tended mainly to differ on the emotional condition (*P* = 0.07). For the N170, no significant main effects were revealed on latency (all *P* > 0.13) nor on amplitude (all *P* > 0.08). No significant interactions were found. The apparent but non-significant N170 group difference (*P* = 0.09) seen on the curves Fig. 2A could be related to the important amplitude variability in the TD group (e.g., emotional and neutral averaged amplitude over PO8: TD: −3.53 ± 5.0 μV, ASD: −1.57 ± 2.5 μV).

### Visual mismatch negativity

Student’s *t* test for each vMMN in each group revealed significant deflections compared to baseline (*P* < 0.05) between 100 and 550 ms.

#### Posterior peak analyses

Visual inspection of potential waveforms over posterior electrodes indicated that two peaks were clearly recognizable in the TD group, occurring at 150 and 310 ms, respectively (see Fig. 2B over PO8 electrode) as previously shown^18^.

In the first time window (80–180 ms), analyses of the posterior peak amplitude revealed an effect of Electrode (*F*(1.71,54.6) = 13.10, *P* < 0.001, *η*^2^_p_ = 0.291) explained by parieto-occipital sites (i.e., PO7 and PO8) presenting more negative responses compared to occipital or parietal electrodes (*P* = 0.009, *P* < 0.001, respectively). A significant interaction between Electrode, Emotion, and Group (*F*(1.87,59.8) = 3.39, *P* = 0.043, *η*^2^_p_ = 0.095) was also found due in particular to the ASD group presenting a smaller vMMN amplitude to the emotional compared to the neutral deviancy over the occipital sites (*P* = 0.012), whereas no emotion effect was found on the first vMMN peak amplitude in the TD group. Latency analyses revealed that responses were faster over the occipital sites compared to parieto-occipital sites (*P* = 0.012) as shown by a significant effect of Electrode (*F*(1.80,57.5) = 4.67, *P* = 0.016, *η*^2^_p_ = 0.127). No group differences or other effects were found in this time window.

In the second time window (220–400 ms), peak analyses of the amplitude revealed a significant Group effect (*F*(1,32) = 5.38, *P* = 0.03, *η*^2^_p_ = 0.144) due to the ASD group presenting a smaller vMMN response. The Electrode effect was also significant (F(1.80,57.7) = 15.83, *P* < 0.001, *η*^2^_p_ = 0.331), as both occipital and parieto-occipital sites presented more negative responses compared to parietal sites (*P* = 0.002, *P* < 0.001, respectively). This effect was further explained by a significant interaction between Group and Electrode (*F*(1.80,57.7) = 5.02, *P* = 0.012, *η*^2^_p_ = 0.136) showing that the Electrode effect was due to the TD group only (both comparisons *P* < 0.001). The Hemisphere effect was also significant (*F*(1,32) = 6.94, *P* = 0.013, *η*^2^_p_ = 0.178) and explained by larger amplitude over the right hemisphere.

Latency analyses revealed a significant effect of the Hemisphere (*F*(1,32) = 5.88, *P* = 0.02, *η*^2^_p_ = 0.155) as the response occurred faster over the left hemisphere. A three-way interaction between the Emotion, Electrode, and Hemisphere was found (*F*(1.94,62) = 3.40, *P* = 0.04, *η*^2^_p_ = 0.096) due to responses occurring faster for the neutral compared to the emotional vMMN over the left (P7) compared to the right (P8) parietal electrode (*P* = 0.002). No significant correlations were found with verbal, non-verbal IQ, or *d*’ in the TD or ASD groups.

#### Mean amplitude analysis of posterior response

Finally, in the 400–480 ms time window, the analysis performed on the vMMN revealed a significant interaction between Group and Emotion (*F*(1,32) = 4.39, *P* = 0.04, *η*^2^_p_ = 0.122), as emotional vMMN response was larger in TD compared to ASD group (*P* < 0.05; emotional response over PO8, TD: −1.15 ± 1.7 μV; ASD: 0.04 ± 1.5 μV). Verbal and non-verbal IQ nor *d*’ correlated with mean vMMN activity.

### Permutation analyses

**Fig. 3:**
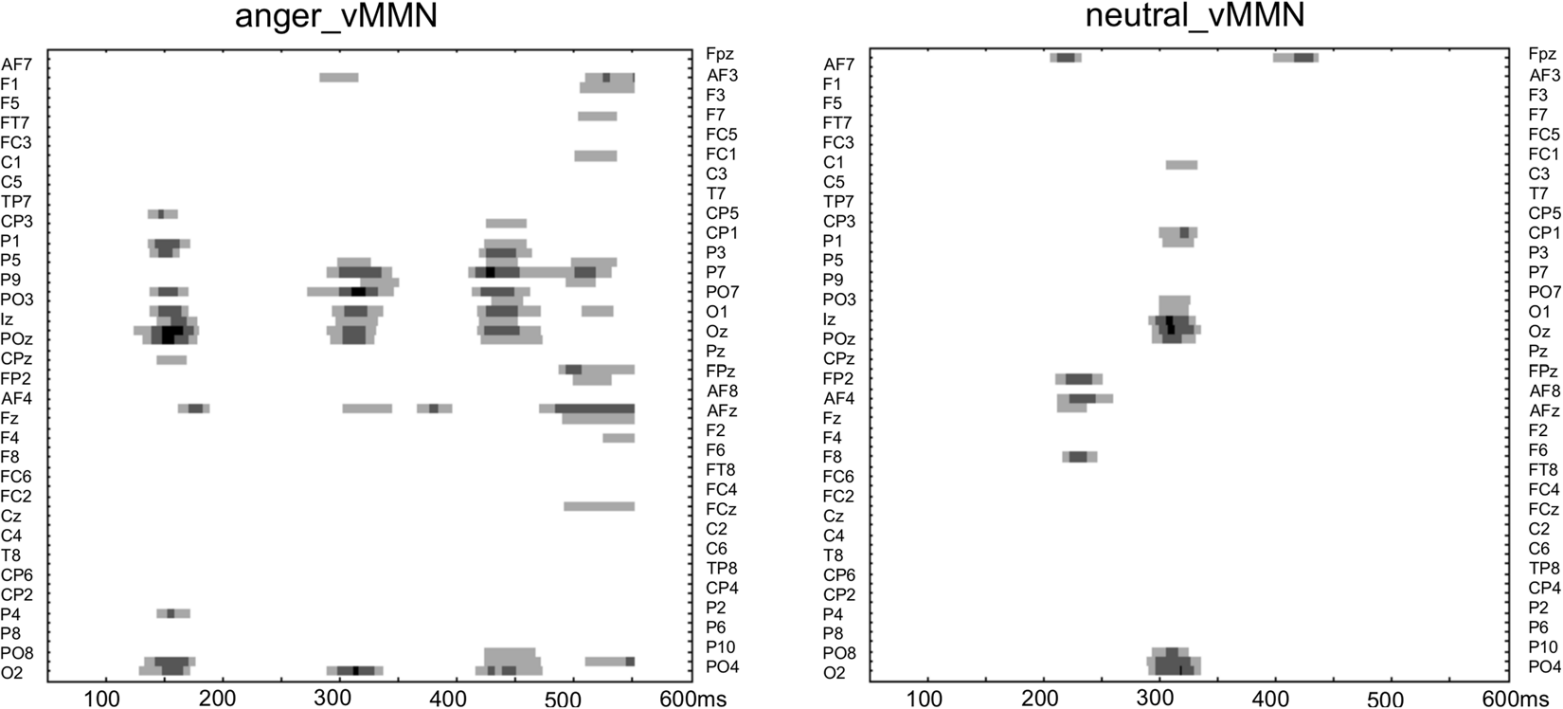
Permutation tests analyses. Permutation tests analyses showing statistical significance between groups over the entire scalp (64 electrodes) in the 50–600 ms latency range for the anger vMMN (left) and the neutralv MMN (right). Statistical significance is represented by light gray, gray, and black colors (*P* < 0.05, *P* < 0.01, and *P* < 0.001, respectively).

As show in Fig. 3, the results showed group differences in mainly three time windows for the emotional condition (anger-MMN), while groups differed only around the 300–320 ms time window for the neutral condition. As no group differences were observed on vMMN peak latencies, permutations confirmed that group differences were due to amplitude differences. These analyses also confirmed the significant Group-by-Emotion interaction found in the mean amplitude analysis in the late time window.

### Source estimations

**Fig. 4:**
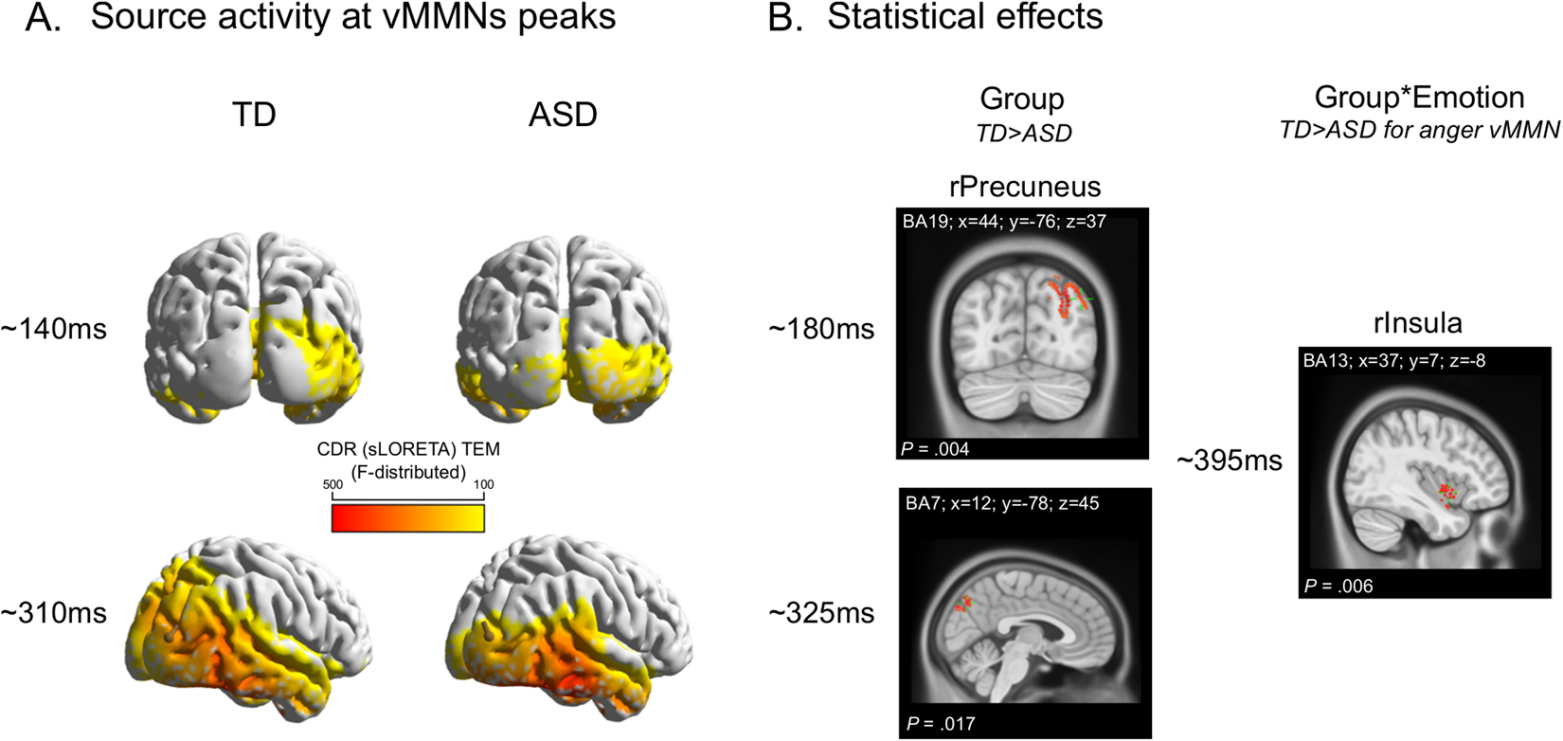
vMMN source activity and group comparison. **A** Source activity for the TD and ASD groups (left and right sides, respectively) around the vMMN peaks (i.e., ~140 ms on the top and ~310 ms on the bottom). **B** Significant statistical effects: on the left side, the group effect in the right precuneus (rPrecuneus) at two latencies (~18 0ms and ~325 ms); on the right, the Group×Emotion interaction, due to right insula (rInsula) being stronger activated in the TD group compared to the ASD group for only the emotional deviancy (i.e., anger_vMMN at ~395 ms).

Source analyses results are summarized in Table 1 and Fig. 4A, B. Table 1 displays brain regions and latencies where significant statistical effects were found.

**Table 1:** CDR statistical analyses.

Comparison	Time window (ms)	Brain region label	Brain region (BA)	MNI coordinates (*x*, *y*, *z*)	*P* value
Anger vs. neutral (Emo effect) Deviant vs. equiprobable (Cond effect) *Comparisons in each group*					
TD					
Cond effect	175–179	lIFG	BA9	−45, 6, 25	*P* = 0.022
	265–285	lSTG	BA22	−66, −43, 8	*P* = 0.001
Emo effect	224–240	rIFG	BA47	33, 26, −7	*P* = 0.006
	299–304	rPrecentral G	BA6	33, −16, 66	*P* = 0.033
	382–406	rlPI	BA40	57, −43, 37	*P* = 0.006
	443–451	rSupraMarginal G	BA40	57, −43, 36	*P* = 0.015
Interaction Emo*Cond	100–107	lSFG	BA10	−38, 49, 20	*P* = 0.009
	156–158	lIPL	BA40	−62, −36, 29	*P* = 0.044
	363–377	rMTG	BA21	70, −22, −14	*P* = 0.003
	415–433	rMOG	BA18	13, −100, 15	*P* = 0.004
		rMFG/rectal	BA11	5, 35, −28	*P* = 0.004
		rACC	BA32	7, 31, −14	*P* = 0.004
ASD					
Cond. effect	189–202	rTP	BA38	22, 7, −40	*P* = 0.004
	248–257	lPrecentral	BA6	−61, −16, 43	*P* = 0.021
	270–277	rInsula	BA13	46, −13, 4	*P* = 0.013
		rSTG	BA22	53, −16, 5	*P* = 0.013
	293–308	lPrecentral	BA6	−37, −9, 63	*P* = 0.001
	375–390	rIPS/precuneus	BA7	28, −57, 54	*P* = 0.022
	417–431	lMFG	BA6	−3, −22, 53	*P* = 0.013
		rMFG	BA6	5, −22, 54	*P* = 0.013
		lCingulate	BA24	−7, −23, 38	*P* = 0.013
		rCingulate	BA24	6, −23, 39	*P* = 0.013
Emo. effect	205–238	lIPL	BA40	−66, −26, 23	*P* = 0.001
Interaction Emo*Cond		No significant effects			
*Group comparisons on vMMNs*					
TD > ASD	171–189	rPrecuneus	BA19	44, −76, 37	*P* = 0.002
	319–341	rPrecuneus	BA7	12, −78, 45	*P* = 0.017
	429–437	lInsula	BA13	−31, 18, −8	*P* = 0.013
	447–454	rMTG	BA39	44, −61, 25	*P* = 0.030
ASD > TD	347–355	rSTG	BA38	44, 17, −37	*P* = 0.034
	429–437	lSTG	BA38	−33, 20, −36	*P* = 0.013
Interaction Group*Emo	392–396	rInsula	BA13	37, 7, −8	*P* = 0.006

### Descriptive analysis

In both groups, CD activity was observed in several brain regions for neutral and emotional vMMN mostly including striate (in ASD) and extrastriate (in TD) occipital cortices in the MMN first peak latency range and the right fusiform gyrus (FG) and right temporal regions during the second MMN peak in both groups (Fig. 4A).

### Within-group statistical analysis

In the TD group, the statistical comparison of CD activity in response to Equiprobable and Deviant stimuli leaded to significant differences at 175 ms in the left inferior frontal gyrus (IFG) and at 275 ms in the left superior temporal gyrus (BA22, STG). In the ASD group, the same analysis displayed significant differences in the right temporal pole (BA38, TP), the right STG/insula, right precuneus, and cingulate regions (Table 1).

Source activities of Neutral and Emotional change detection were then directly compared in the Emotion (Anger vs. Neutral) by Condition (Equiprobable vs. Deviant) interaction within each group. The analysis in the TD group showed greater activity for the Emotional than for the Neutral change detection in the left superior frontal gyrus (BA10), left inferior parietal lobule (IPL) (BA40), right middle temporal gyrus (MTG), and later in the right cuneus, right middle frontal gyrus/rectal (BA11) and right anterior cingulate cortex. In the ASD group, no interaction was observed between Emotion (Anger vs. Neutral) and Condition (Equiprobable vs. Deviant) in any of the time windows.

### Between-group statistical analysis

Group comparison of vMMN sources revealed in the ASD group a significant lower activity compared to the TD group in early time windows in the Precuneus (BA7, 19) and later in the insula (BA13) and in the posterior MTG (BA39) but larger activity in the TP (BA38) in a later time window (340–440 ms). Moreover, significant Group-by-Emotion interaction (i.e., Anger and neutral vMMN) (*P* = 0.002) revealed reduced duration of the source activity in the right insula during emotional change compared to neutral change detection at 395 ms time window in the ASD group, whereas source in the insula maintained its activity for emotional change in the TD group (Table 1 and Fig. 4B).

## Discussion

By using both oddball and equiprobable sequences, we investigated specific automatic change detection of emotional (i.e., anger) and neutral deviant faces in adults with autism. To our knowledge, this is the first study characterizing automatic deviancy processing to facial expressions in autism by investigating visual MMN and its brain sources.

From the early stages of sensory perception, our results showed atypical responses to faces in individuals with ASD. Indeed, as in a previous study, P100 amplitude was found reduced to emotional face stimuli^7^, in line with early visual responses being affected in those with ASD^43–45^. Conversely, N170 amplitude did not differ between the groups as recently shown in a meta-analysis in ASD^46^. Moreover, we showed that only the TD group displayed a greater response to the angry stimulus compared to the neutral one, while this modulation by the emotion was not observed in the ASD group^7,47^. These atypical sensory responses suggest that early steps of face and facial expression processing are altered in adults with ASD, in accordance with previous neuroimaging findings showing atypical responses in the occipito-temporal regions^6,48–50^. As demonstrated by the increasing number of studies using vMMN paradigms in psychiatric neurodevelopmental and neurodegenerative conditions, vMMN component is considered as a potential electrophysiological marker of automatic change detection (for a review and meta-analysis, see ref. ^21^). However, to date only few studies have specifically investigated facial expression-related vMMN in clinical populations^27–29,51,52^.

In the present study, similarly to previous investigations on facial expression vMMN, two peaks were observed in the TD group around 150 and 310 ms^17,18,27,53^, with the second peak being followed by a sustained activity for the emotional deviant stimulus but not for the neutral one (see ref. ^18^). Analyses of vMMN peaks revealed that change detection responses were different in the ASD and TD groups in the earliest time window (80–180 ms) as only the ASD group presented a reduced emotional response, while the TD group shows similar responses for the neutral and the angry deviants. Reduced vMMN was observed on the second peak in the 220–400 ms latency range in response regardless of the nature of the deviant stimuli. This corroborates previous studies investigating MMN in the visual modality in ASD using simple non-social stimuli and showing disrupted change detection processing in both children and adults^9,23^. It is possible that individuals with ASD favor low-level information at the expense of relevant features influenced by the context^54,55^. This could turn into a smaller vMMN amplitude compared to TD adults, especially in the case of a controlled vMMN where the stimulus presented in the equiprobable sequence is subtracted from the same stimulus presented as deviant in the oddball sequence allowing to control for low-level features. Conversely, TD individuals might rely more on higher-level information depending on the context (i.e., sequence in which the stimulus is presented), than on low-level aspects while automatically processing changing events (see ref. ^54^).

Such atypical change detection in participants with ASD has also been shown in the auditory modality in response to both physical (tone frequency)^10,11^ and prosodic (emotional) deviancies^56^. Altogether, results from visual and auditory MMN studies support the hypothesis of a broad impairment of the automatic change detection system.

In accordance with our expectations, we found no emotional sustained activity in the ASD group in the later time window as compared to TD participants^18^. Overall, group differences point for an impairment of change detection mechanism as well as for a specific emotional change detection deficit. This was also confirmed by permutation analyses showing that group differences were found for the emotional condition around an earlier time window compared to the neutral condition. Thus, emotional deviant events may not be processed as being particularly salient as in TD adults and might be under-processed in participants with ASD, leading to major difficulties in social adaptation. Altogether, these results are in line with the positive correlation between AQ scores measured in TD adults and vMMN amplitude to happy deviant stimuli as previously shown^30^, meaning that more autistic traits were associated with smaller vMMN amplitude (i.e. more positive).

In order to better understand the brain processes involved in facial expression change detection, exploratory and complementary source analyses were performed. Only few studies investigated sources in emotional vMMN in TD participants^15,16,28,37^, and this is the first to investigate source activity of vMMN responses to emotional and neutral deviants in people with ASD. Foremost, in both groups, we replicated previous results reported in TD populations and found activity in the insula and in the occipital and temporal cortices (i.e., STG, FG, see refs. ^16,37^) during the vMMN process to change in facial expressions. Hence, the broad face processing network was involved regardless of the emotional content in both groups, suggesting that when gaze is forced to a specific face area (nearby the eyes’ region as in our protocol), the FG is typically activated in autistic individuals^48,57^. Second, we showed that during change processing, compared to controls, individuals with autism displayed reduced activity in the posterior MTG/IPL (BA 19/39), regions belonging to the automatic saliency network^58^. Results also indicated reduced activity in the insula in the ASD group while processing the emotional deviancy specifically. Here again, findings suggest that the activity of the brain regions involved in the broad change detection process is atypical and also not modulated by the emotional nature of the stimuli. By showing reduced activity of the insula in those with ASD, the present results are in accordance with a meta-analysis showing a hypo-activation of this region during face perception^59^. However, such hypo-activation was also observed in non-social cognition contexts, in line with the insula contributing to the processing of saliency and also integrating together sensory and affective information^60^. Other studies, however, show a hyper-activation of this region in autism, especially in children (for a review, see ref. ^60^), suggesting that, depending on the specific process and age group, different patterns can be observed. In line with this, our results suggest an impaired activity of the insula related to atypical change detection of emotional faces, which might support that this region plays a critical role in both affective and pre-attentional processes.

Some limitations should be considered in the present study. To our knowledge, this is the first investigation of vMMN to emotional face in adults with autism, which requires to be further replicated. These data should be confirmed within a larger sample size and by using other emotional facial expressions. vMMN protocols often require the participants to be involved in an active concurrent task, therefore constraining this kind of investigation to the higher part of the autistic spectrum. Other protocols should be designed to allow studying automatic change detection within the visual modality in individuals from the whole autistic spectrum. In a similar vein, studying the vMMN to facial expressions in younger participants could provide crucial information on the developmental aspects of the automatic detection of emotional changes. Finally, since similar atypical change detection to emotional faces was found in other clinical conditions such as schizophrenia, it would be of interest to compare different groups to investigate whether our results are specific to the ASD population.

## Conclusions

The present study provides additional understanding of the automatic emotional processing in autism. Here we combine both understanding of atypical emotional face processing and unusual automatic change detection in autism (see ref. ^61^). Indeed, these findings suggest that both atypical tracking of subtle changes and less accurate processing of facial expressions could be responsible for the difficulties in ASD to adapt to complex and rapidly changing social situations.

## Data Availability

Data supporting the findings of this study are available from the corresponding author upon request.

## References

[1] APA. *Diagnostic and Statistical Manual of Mental Disorders (DSM-5)* (American Psychiatric Association, Washington, DC, 2013).

[2] Gomot M, Wicker B (2012). A challenging, unpredictable world for people with autism spectrum disorder. Int. J. Psychophysiol..

[3] Harms MB, Martin A, Wallace GL (2010). Facial emotion recognition in autism spectrum disorders: a review of behavioral and neuroimaging studies. Neuropsychol. Rev..

[4] Evers K, Steyaert J, Noens I, Wagemans J (2015). Reduced recognition of dynamic facial emotional expressions and emotion-specific response bias in children with an autism spectrum disorder. J. Autism Dev. Disord..

[5] Black MH (2017). Mechanisms of facial emotion recognition in autism spectrum disorders: Insights from eye tracking and electroencephalography. Neurosci. Biobehav. Rev..

[6] Critchley HD (2000). The functional neuroanatomy of social behaviour: changes in cerebral blood flow when people with autistic disorder process facial expressions. Brain.

[7] Batty M, Meaux E, Wittemeyer K, Roge B, Taylor MJ (2011). Early processing of emotional faces in children with autism: an event-related potential study. J. Exp. Child Psychol..

[8] Clery H, Andersson F, Fonlupt P, Gomot M (2013). Brain correlates of automatic visual change detection. Neuroimage.

[9] Clery H (2013). Atypical visual change processing in children with autism: an electrophysiological study. Psychophysiology.

[10] Gomot M (2011). Candidate electrophysiological endophenotypes of hyper-reactivity to change in autism. J. Autism Dev. Disord..

[11] Gomot M, Giard MH, Adrien JL, Barthelemy C, Bruneau N (2002). Hypersensitivity to acoustic change in children with autism: electrophysiological evidence of left frontal cortex dysfunctioning. Psychophysiology.

[12] Gomot M (2006). Change detection in children with autism: an auditory event-related fMRI study. Neuroimage.

[13] Kimura M, Schroger E, Czigler I (2011). Visual mismatch negativity and its importance in visual cognitive sciences. Neuroreport.

[14] Qian X (2014). The visual mismatch negativity (vMMN): toward the optimal paradigm. Int. J. Psychophysiol..

[15] Kimura M, Kondo H, Ohira H, Schroger E (2012). Unintentional temporal context-based prediction of emotional faces: an electrophysiological study. Cereb. Cortex.

[16] Vogel BO, Shen C, Neuhaus AH (2015). Emotional context facilitates cortical prediction error responses. Hum. Brain Mapp..

[17] Astikainen P, Cong F, Ristaniemi T, Hietanen JK (2013). Event-related potentials to unattended changes in facial expressions: detection of regularity violations or encoding of emotions?. Front. Hum. Neurosci..

[18] Kovarski K (2017). Facial expression related vmmn: disentangling emotional from neutral change detection. Front. Hum. Neurosci..

[19] Rosburg T, Weigl M, Deuring G (2019). Enhanced processing of facial emotion for target stimuli. Int. J. Psychophysiol..

[20] Kuehne M, Siwy I, Zaehle T, Heinze HJ, Lobmaier JS (2019). Out of focus: facial feedback manipulation modulates automatic processing of unattended emotional faces. J. Cogn. Neurosci..

[21] Kremlacek J (2016). Visual mismatch negativity (vMMN): a review and meta-analysis of studies in psychiatric and neurological disorders. Cortex.

[22] Clery H (2013). fMRI investigation of visual change detection in adults with autism. Neuroimage Clin..

[23] Clery H (2013). Electrophysiological evidence of atypical visual change detection in adults with autism. Front. Hum. Neurosci..

[24] Kemner C, Verbaten MN, Cuperus JM, Camfferman G, Van Engeland H (1994). Visual and somatosensory event-related brain potentials in autistic children and three different control groups. Electroencephalogr. Clin. Neurophysiol..

[25] Sokhadze E (2009). Event-related potential study of novelty processing abnormalities in autism. Appl. Psychophysiol. Biofeedback.

[26] Maekawa T (2011). Top-down and bottom-up visual information processing of non-social stimuli in high-functioning autism spectrum disorder. Res. Autism Spectr. Disord..

[27] Chang Y, Xu J, Shi N, Zhang B, Zhao L (2010). Dysfunction of processing task-irrelevant emotional faces in major depressive disorder patients revealed by expression-related visual MMN. Neurosci. Lett..

[28] Csukly G, Stefanics G, Komlosi S, Czigler I, Czobor P (2013). Emotion-related visual mismatch responses in schizophrenia: impairments and correlations with emotion recognition. PLoS ONE.

[29] Tang D (2013). Visual mismatch negativity in the detection of facial emotions in patients with panic disorder. Neuroreport.

[30] Gayle LC, Gal DE, Kieffaber PD (2012). Measuring affective reactivity in individuals with autism spectrum personality traits using the visual mismatch negativity event-related brain potential. Front. Hum. Neurosci..

[31] Dichter GS, Felder JN, Bodfish JW (2009). Autism is characterized by dorsal anterior cingulate hyperactivation during social target detection. Soc. Cogn. Affect Neurosci..

[32] Schroger E, Wolff C (1996). Mismatch response of the human brain to changes in sound location. Neuroreport.

[33] Kimura M, Katayama J, Ohira H, Schroger E (2009). Visual mismatch negativity: new evidence from the equiprobable paradigm. Psychophysiology.

[34] Lord C (2000). The autism diagnostic observation schedule-generic: a standard measure of social and communication deficits associated with the spectrum of autism. J. Autism Dev. Disord..

[35] Lord C, Rutter M, Le Couteur A (1994). Autism Diagnostic Interview-Revised: a revised version of a diagnostic interview for caregivers of individuals with possible pervasive developmental disorders. J. Autism Dev. Disord..

[36] Wechsler, D. *Wechsler Intelligence Scale for Children (Fourth Edition, WISC-IV)* (The Psychological Corporation, San Antonio, TX, 2005).

[37] Li X, Lu Y, Sun G, Gao L, Zhao L (2012). Visual mismatch negativity elicited by facial expressions: new evidence from the equiprobable paradigm. Behav. Brain Funct..

[38] Jacobsen T, Schroger E (2001). Is there pre-attentive memory-based comparison of pitch?. Psychophysiology.

[39] Aguera PE, Jerbi K, Caclin A, Bertrand O (2011). ELAN: a software package for analysis and visualization of MEG, EEG, and LFP signals. Comput. Intell. Neurosci..

[40] Guthrie D, Buchwald JS (1991). Significance testing of difference potentials. Psychophysiology.

[41] Fuchs M, Kastner J, Wagner M, Hawes S, Ebersole JS (2002). A standardized boundary element method volume conductor model. Clin. Neurophysiol..

[42] Wagner M, Ponton C, Tech R, Fuch M, Kastner J (2014). Non-parametric statistical analysis of EEG/MEG map topographies and source distributions on the epoch level. Hum. Cogn. Neurophysiol..

[43] Kovarski K (2016). Brief report: early VEPs to pattern-reversal in adolescents and adults with autism. J. Autism Dev. Disord..

[44] Pei, F., Baldassi, S. & Norcia, A. M. Electrophysiological measures of low-level vision reveal spatial processing deficits and hemispheric asymmetry in autism spectrum disorder. *J. Vis.* **14**, 3 (2014). 10.1167/14.11.3 25194015

[45] Kornmeier J, Worner R, R A, Bach M, vE LT (2014). A different view on the checkerboard? Alterations in early and late visually evoked EEG potentials in asperger observers. PLoS ONE.

[46] Kang E (2018). Atypicality of the N170 event-related potential in autism spectrum disorder: a meta-analysis. Biol. Psychiatry.

[47] de Jong MC, van Engeland H, Kemner C (2008). Attentional effects of gaze shifts are influenced by emotion and spatial frequency, but not in autism. J. Am. Acad. Child Adolesc. Psychiatry.

[48] Hadjikhani N (2004). Activation of the fusiform gyrus when individuals with autism spectrum disorder view faces. Neuroimage.

[49] Pelphrey KA (2002). Visual scanning of faces in autism. J. Autism Dev. Disord..

[50] Kovarski K (2019). Enhanced early visual responses during implicit emotional faces processing in autism spectrum disorder. J. Autism Dev. Disord..

[51] Xu Q (2018). Automatic processing of changes in facial emotions in dysphoria: a magnetoencephalography study. Front. Hum. Neurosci..

[52] She S (2017). Revealing the dysfunction of schematic facial-expression processing in schizophrenia: a comparative study of different references. Front. Neurosci..

[53] Astikainen P, Hietanen JK (2009). Event-related potentials to task-irrelevant changes in facial expressions. Behav. Brain Funct..

[54] Pellicano E, Burr D (2012). When the world becomes ‘too real’: a Bayesian explanation of autistic perception. Trends Cogn. Sci..

[55] Mottron L, Dawson M, Soulieres I, Hubert B, Burack J (2006). Enhanced perceptual functioning in autism: an update, and eight principles of autistic perception. J. Autism Dev. Disord..

[56] Charpentier J (2018). Emotional prosodic change detection in autism spectrum disorder: an electrophysiological investigation in children and adults. J. Neurodev. Disord..

[57] Perlman SB, Hudac CM, Pegors T, Minshew NJ, Pelphrey KA (2011). Experimental manipulation of face-evoked activity in the fusiform gyrus of individuals with autism. Soc. Neurosci..

[58] Corbetta M, Kincade JM, Ollinger JM, McAvoy MP, Shulman GL (2000). Voluntary orienting is dissociated from target detection in human posterior parietal cortex. Nat. Neurosci..

[59] Di Martino A (2009). Functional brain correlates of social and nonsocial processes in autism spectrum disorders: an activation likelihood estimation meta-analysis. Biol. Psychiatry.

[60] Nomi JS, Molnar-Szakacs I, Uddin LQ (2019). Insular function in autism: update and future directions in neuroimaging and interventions. Prog. Neuropsychopharmacol. Biol. Psychiatry.

[61] Latinus M (2019). Inflexibility in autism spectrum disorder: need for certainty and atypical emotion processing share the blame. Brain Cogn..

